# Evaluation of Taxonomic Characteristics of Matlo and Phala Bat Rabies-Related Lyssaviruses Identified in South Africa

**DOI:** 10.3390/v15102047

**Published:** 2023-10-04

**Authors:** Natalie Viljoen, Jacqueline Weyer, Jessica Coertse, Wanda Markotter

**Affiliations:** 1 Centre for Viral Zoonoses, Department of Medical Virology, University of Pretoria, Pretoria 0001, South Africa; 2 Centre for Emerging Zoonotic and Parasitic Diseases, National Institute for Communicable Disease of the National Health Laboratory Service, Johannesburg 2131, South Africa; 3 Department of Microbiology and Infectious Diseases, Faculty of Health Sciences, University of Witwatersrand, Johannesburg 2000, South Africa

**Keywords:** lyssavirus, rabies, South Africa, bat, surveillance, molecular characterization

## Abstract

We report the genetic characterization of two potentially novel rabies-related lyssaviruses identified from bats in Limpopo province, South Africa. Matlo bat lyssavirus (MBLV) was identified in two *Miniopterus natalensis* (Natal long-fingered) bats in 2015 and 2016, and Phala bat lyssavirus (PBLV) was identified in a *Nycticeinops schlieffeni* (Schlieffen’s) bat in 2021. The distribution of both of these bat species is largely confined to parts of Africa, with limited reports from the Arabian Peninsula. MBLV and PBLV were demonstrated to group with the unassigned and phylogroup I lyssaviruses, respectively. MBLV was most closely related to *Lyssavirus caucasicus* (WCBV), whereas PBLV was most closely related to *Lyssavirus formosa* (TWBLV-1) and Taiwan bat lyssavirus 2 (TWBLV-2), based on analysis of the N and G genes, the concatenated N + P + M + G + L coding sequence, and the complete genome sequence. Based on our analysis, MBLV and WCBV appeared to constitute a phylogroup separate from *Lyssavirus lleida* (LLEBV) and *Lyssavirus ikoma* (IKOV). Analysis of the antigenic sites suggests that PBLV will likely be serologically distinguishable from established lyssaviruses in virus-neutralization tests, whereas MBLV appeared to be antigenically highly similar to WCBV. Taken together, the findings suggested that, while PBLV is likely a new lyssavirus species, MBLV is likely related to WCBV.

## 1. Introduction

Lyssaviruses are enveloped, bullet-shaped negative-sense single-stranded RNA viruses that belong to the genus *Lyssavirus*, subfamily *Alpharhabovirinae*, family *Rhabodoviridae* [1,2]. The viral genome is approximately 12 kb in length and encodes five proteins, which are all multifunctional and include the nucleo- (N), phospho- (P), matrix- (M), glycoprotein (G), and RNA-dependent RNA polymerase (L), reviewed in [3]. Among lyssaviruses, the N gene is the most conserved [4]. Lyssaviruses are phylogenetically grouped into two phylogroups; however, there are lyssaviruses that do not group within phylogroups I or II and remain unassigned. Although all lyssaviruses are capable of causing rabies, an acute progressive encephalomyelitis, lyssaviruses that belong to different phylogroups have distinct characteristics, including differences in their pathogenesis, immunogenicity, and the degree of cross-neutralization [5]. A recent study that assessed the pathogenicity of phylogroup I lyssaviruses using a standardized intramuscular pathogenicity index (IMPI) score suggested that *Lyssavirus irkut* (IRKV) and *Lyssavirus bokeloh* (BBLV) may be more pathogenic than *Lyssavirus rabies* (RABV), challenging the suggestion that non-RABV bat lyssaviruses are less pathogenic than RABV [6].

The International Committee on Taxonomy of Viruses (ICTV) report for the family *Rhabdoviridae* released in 2022 recognized 17 lyssaviruses and listed 1 putative species, Kotalahti bat lyssavirus (KBLV), which awaits formal classification [2]. In addition, several potentially novel lyssaviruses have been described, including Matlo bat lyssavirus (MBLV), identified in two apparently healthy *Miniopterus natalensis* (Natal long-fingered) bats in South Africa [7,8], Taiwan bat lyssavirus 2 (TWBLV-2), identified from a dead *Nyctalus plancyi velutinus* (Chinese noctule) bat in Taiwan [9], and Phala bat lyssavirus (PBLV), identified from a dead *Nycticeinops schlieffeni* (Schlieffen’s) bat in South Africa [10]. The genetic characterization of two lyssaviruses that are closely related to previously described lyssaviruses was reported. Ozernoe lyssavirus was reported in a human rabies case after exposure to an unidentified bat and was shown to be closely related to IRKV [11], whereas Divača bat lyssavirus (DLBV) was identified in a dead *Myotis capaccinii* bat in Slovenia during a retrospective surveillance program and was shown to be closely related to KBLV [12]. 

Bats are the natural reservoir hosts for lyssaviruses, with the exception of *Lyssavirus mokola* (MOKV), identified in wild-caught shrews (*Crocidura flavescens manni*) [13], and *Lyssavirus ikoma* (IKOV), identified in an African civet (*Civettictis civetta*) [14]; however, these infections were likely due to cross-species transmission, and it is unlikely that these terrestrial animal species are the reservoir hosts for these lyssaviruses. The majority of lyssaviruses appear to have co-evolved with a limited number of reservoir species and are geographically restricted, except RABV [15]. In contrast to the rabies-related lyssaviruses, RABV has global distribution, except for a few territories that are free from terrestrial rabies, and it is reported in bats only in the Americas and is well-established in various terrestrial animal species [15]. Continuous surveillance of bat populations is required to improve our understanding of the epidemiology of lyssaviruses, to identify novel and/or emerging lyssaviruses, and to identify, assess, and mitigate the risk to animal and human health. The majority of established and proposed lyssaviruses have been discovered through active or passive bat surveillance, including *Lyssavirus aravan* (ARAV) [16], *Lyssavirus australis* (ABLV) [17], BBLV [18], *Lyssavirus caucasicus* (WCBV) [19], *Lyssavirus formosa* (TWBLV-1) [20], TWBLV-2 [9], *Lyssavirus gannoruwa* (GBLV) [21], *Lyssavirus hamburg* (EBLV-1) [22], IRKV [19], *Lyssavirus khujand* (KHUV) [23], *Lyssavirus lagos* (LBV) [24], *Lyssavirus lleida* (LLEBV) [25], KBLV [26], MBLV [7], PBLV [10], and *Lyssavirus shimoni* (SHIBV) [27], whereas the first described cases of *Lyssavirus duvenhage* (DUVV) [28] and *Lyssavirus helsinki* (EBLV-2) [29] were in humans who developed rabies after bat exposures.

With enhanced bat surveillance and virus discovery efforts, a growing diversity of lyssaviruses has been described; however, it is important to note that bat surveillance is still inadequate in large parts of the world. Despite inadequate lyssavirus surveillance in Africa, a diversity of lyssaviruses has been described, which may not represent the true lyssavirus diversity on the continent. In addition, the geographical distribution, genetic diversity, and host-species associations that inform potential mitigation efforts are poorly understood, which may, in part, be due to the rarity of rabies surveillance programs in Africa that further characterize positive results. The gold standard diagnostic test used in rabies surveillance programs, a fluorescent antibody test, relies on the use of an anti-RABV conjugate that detects conserved antigenic sites on the RABV N protein and cannot be used for lyssavirus species differentiation. Therefore, without further characterization using monoclonal antibody typing or sequencing, the results are reported as RABV infections and do not only contribute to the aforementioned shortcomings in our knowledge of rabies-related viruses but may also result in the underestimation of the prevalence of rabies-related virus infections. 

In this paper, we describe the genetic characterization of two potentially novel lyssaviruses, MBLV and PBLV, identified in bats in South Africa, and evaluate the ecological niche of the bat species in which MBLV and PBLV were identified. Our findings suggest that PBLV is likely a new lyssavirus, while MBLV is related to WCBV. MBLV was identified during routine biosurveillance activities and detected in two apparently healthy *M. natalensis* bats collected from the Matlapitsi and Madimatle caves in 2015 and 2016, respectively, in Limpopo province, South Africa, as previously described [7,8]. PBLV was detected in an *N. schlieffeni* bat with neurological signs submitted for further evaluation after it died within 24 h of being submitted to a wildlife rehabilitation center in Phalaborwa, Limpopo province, South Africa, in 2021, as previously described [10]. 

## 2. Materials and Methods

### 2.1. Genome Characterization

The complete genome or coding-complete sequence data were obtained for representative and newly described lyssaviruses from GenBank, including RABV PV-2061 (JX276550.1), RABV (NC001542.1), GBLV (NC031988.1), ABLV (NC003243.1), KBLV (LR994545.1), EBLV-2 (NC009528.2), KHUV (NC025385.1), BBLV (NC025251.1), ARAV (NC020808.1), IRKV (NC020809.1), EBLV-1 (NC009527.1), DUVV (NC020810.1), TWBLV-1 (NC055474.1), TWBLV-2 (ON437589.1), PBLV (OQ970171.1), LBV (NC020807.1), MOKV (NC006429.1), SHIBV (NC025365.1), IKOV (NC018629.1), LLEBV (NC031955.1), WCBV (NC025377.1), and MBLV (MW653808.1). The coding and intergenic regions were identified using a combination of sequence alignment using Muscle [30] in MEGA V11.0.11 [31] and genome annotation using BLAST [32], available at https://blast.ncbi.nlm.nih.gov/Blast.cgi (accessed on 18 June 2023). The nucleotide and amino acid identities were determined for the N, P, M, G, and L genes, and concatenated N + P + M + G + L coding sequences (CDS) using Clustal Omega [33], available at https://www.ebi.ac.uk/Tools/msa/clustalo/ (accessed on 29 August 2023). Important pathogenic and antigenic determinants were identified in the translated and aligned N, P, M, G, and L CDS and compared.

### 2.2. Phylogenetic Analysis

All phylogenetic analyses were performed using CIPRES on XSEDE [34]. The best-fit model of nucleotide substitution for phylogenetic analysis was determined using the Bayesian information criterion in JModelTest2 V2.1.10 [35]. Bayesian inference was used to infer phylogeny using the N gene, G gene, concatenated N + P + M + G + L CDS, and complete genome sequences of representative members of the genus *Lyssavirus*. The best-fit model, a general time-reversible substitution model with invariant sites and gamma distribution, was employed for each dataset with BEAST2 V2.7.3 [36] using the JModelTest2 output. An underlying coalescent process with constant population size and Markov chain Monte Carlo (MCMC) chains of 50 million generations was assumed. The MCMC trace files were visualized and analyzed using Tracer V1.7.2 [37], and the best-fit tree was identified with a burn-in of 10% using TreeAnnotator V2.7.5 [36]. Phylogenetic trees were rendered using Interactive Tree of Life (ITOL) [38], available at https://itol.embl.de/upload.cgi (accessed on 30 August 2023). 

### 2.3. Antigenic Distance Estimation

The antigenic distance estimates were calculated as previously described [39]. Briefly, the translated amino acid sequences of the lyssavirus G protein were aligned using Muscle [30] in MEGA V11.0.11 [31]. The Hamming distances between all lyssavirus sequences were compiled in an Euclidean matrix. The Hammington distance between two lyssaviruses was calculated using the sum of scores for all antigenic sites divided by the number of antigenic sites. The score for each antigenic site was calculated by dividing the number of amino acid changes by the total number of amino acids and multiplying it by 20. A heatmap was created using the calculated Hamming distances in Heatmapper, available at http://www.heatmapper.ca/pairwise/ (accessed on 16 August 2023), using the calculate distance matrix function. A score of zero implies that the two viruses evaluated had identical antigenic sites, whereas more dissimilar antigenic sites were represented by an increase in the score.

### 2.4. Evaluation of Ecological Niche

The ecological niches of *M. natalensis* (A. Smith, 1833) [40] and *N. schlieffeni* (Peters, 1859) [41] were determined by examining the existing literature and mapping occurrence data obtained from the Global Biodiversity Information Facility (GBIF), available at https://www.gbif.org/ (accessed on 14 and 24 May 2023) [42,43]. A total of 1238 occurrence records were available for *M. natalensis*; however, only 888 were georeferenced records, of which 4 records were excluded due to invalid coordinates. A total of 507 occurrence records were available for *N. schlieffeni*; however, only 277 were georeferenced records, of which 4 records were excluded due to invalid coordinates. The mapping of *M. natalensis* and *N. schlieffeni* distribution was performed using QGIS V3.28.2 [44] with the Google hybrid layer, available at http://mt0.google.com/vt/lyrs=y&hl=en&x={x}&y={y}&z={z} (accessed on 24 March 2023). The distribution of the *Miniopterus schreibersii* (Natterer, 1819; Schreibers’ long-fingered), *Pipistrellus abramus* (Temminck, 1838; Japanese pipistrelle), and *N. p. velutinus* (Allen, 1923; Chinese noctule) bats were mapped to determine if any overlap in the distribution of the host species of the most closely related lyssaviruses to MBLV and PBLV exist, as described for *M. natalensis* and *N. schlieffeni* [45,46,47]. Of the 22134, 3402, and 95 occurrence records available for *M. schreibersii*, *P. abramus*, and *N. p. velutinus*, only 21,781, 1589, and 28 were georeferenced records, respectively. All records with invalid coordinates were excluded.

## 3. Results

### 3.1. Genome Organization and Characteristics

**Figure 1:**
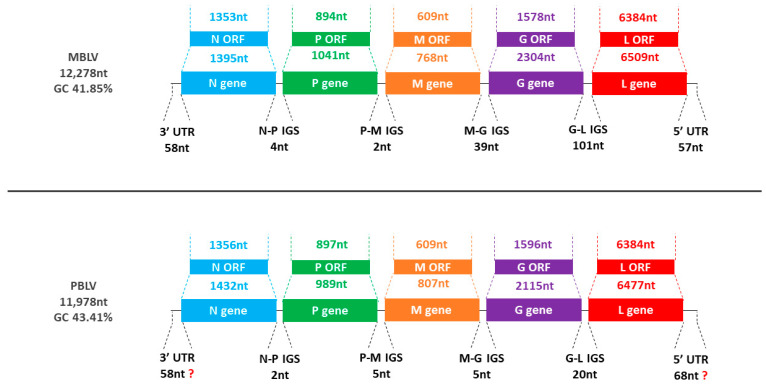
Representation of MBLV and PBLV genome organization. The schematic representation illustrates the genome size, GC content, gene order (N-P-M-G-L), and the length of each gene, open reading frame (ORF), intergenic sequence (IGS), and untranslated region (UTR).

The MBLV genome was 12,278 nucleotides in length and had a GC content of 41.85%, whereas the PBLV genome was 11,978 nucleotides in length and had a GC content of 43.41%. The genome organization of MBLV and PBLV was consistent with known lyssaviruses (Table 1, Figure 1). The transcription initiation signals (TIS) and transcription terminal signals (TTS) are provided (Table 2).

The nucleotide and amino acid identities suggest that MBLV was most closely related to WCBV, an unassigned lyssavirus, with 77.52%, 70.11%, and 78.94% nucleotide identities and 95.56%, 86.67%, and 91.64% amino acid identities of the N gene, G gene, and concatenated N + P + M + G + L CDS, respectively (Table S1). The nucleotide and amino acid identities suggested that PBLV was most closely related to EBLV-1, a phylogroup I lyssavirus, with 74.56%, 65.49%, and 75.55% nucleotide identities and 92.46%, 74.62%, and 86.92% amino acid identities of the N gene, G gene, and concatenated N + P + M + G + L CDS, respectively (Table S2). 

The evaluation of the primer binding site targeted by the lys001 primer (5′-ACGCTTAACGAMAAA-3′) of PBLV revealed that it was significantly different from that of other lyssaviruses (5′-TTGTTTAACAACAAA-3′). In addition, despite the alignment of PBLV with previously described complete lyssavirus genomes suggesting that the complete genome was determined, end verification sequence data suggest that the ends may be longer than reported [10], hence the reporting of the coding-complete and not-complete genome for PBLV. 

### 3.2. Phylogenetic Analysis

**Figure 2:**
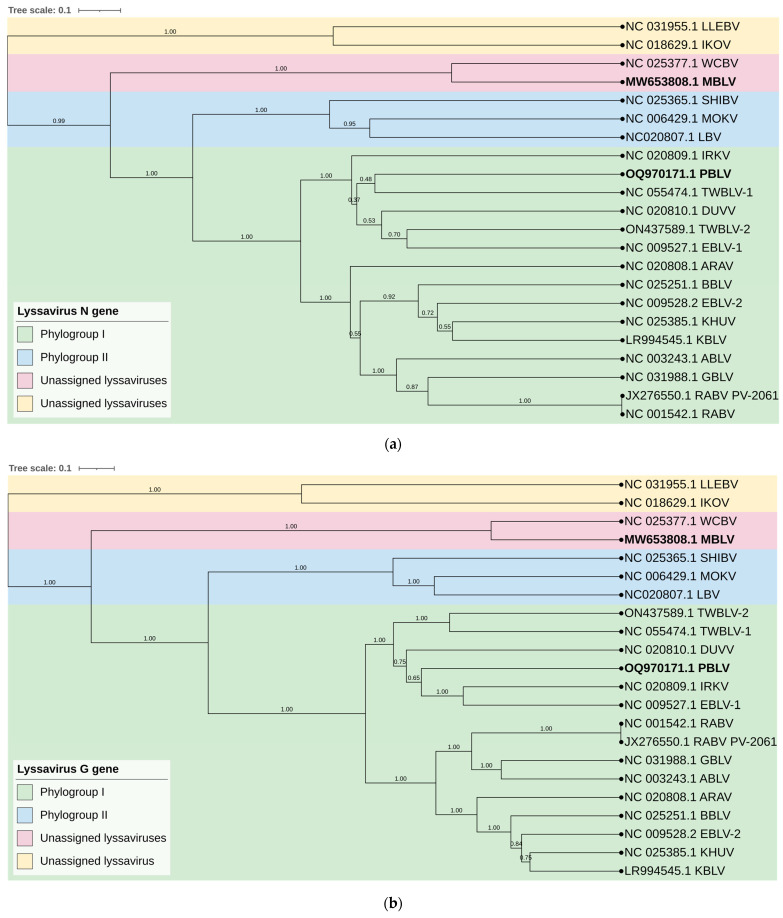

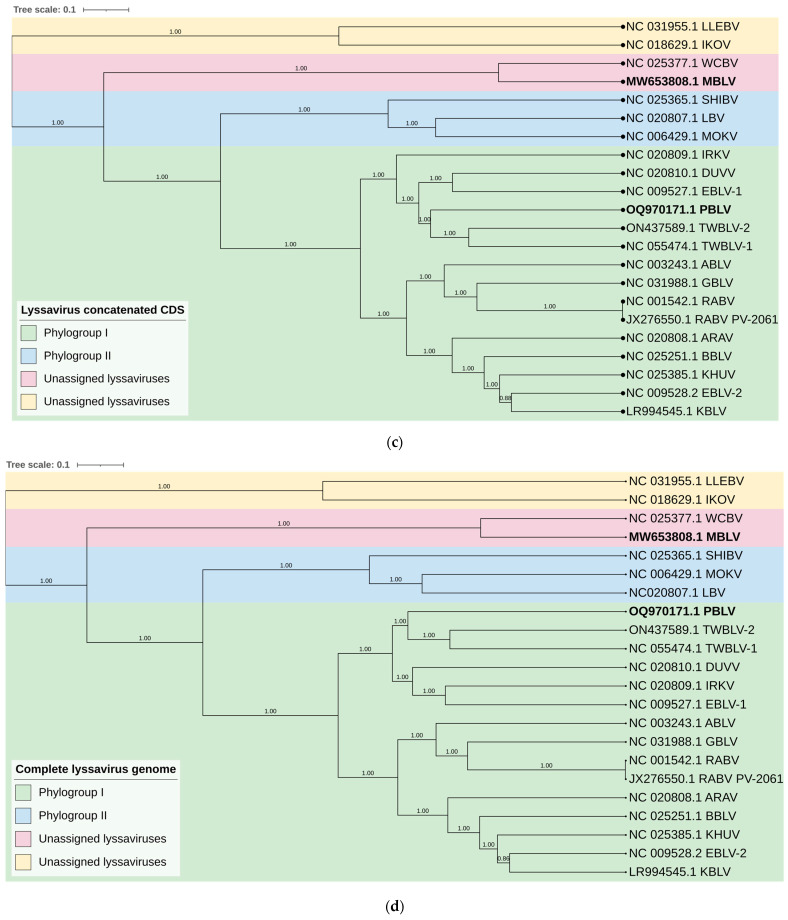
Bayesian analysis of the N and G genes, N + P + M + G + L CDS, and complete genome sequences. Phylogenetic trees were inferred based on the nucleotide sequences of the (**a**) N gene, (**b**) G gene, (**c**) concatenated N + P + M + G + L CDS, and (**d**) complete genome sequences. The Bayesian posterior probabilities are indicated for each node. Clades are highlighted in different colors to illustrate the grouping of phylogroups I and II and unassigned lyssaviruses. The unassigned lyssaviruses were represented in two separate clades in all phylogenetic trees (posterior probability of 1.0), which suggests that the unassigned lyssaviruses may constitute two phylogroups.

Phylogenetic analysis confirmed that MBLV and PBLV group with the unassigned and phylogroup I lyssaviruses, respectively. All phylogenetic trees infer that MBLV and WCBV were closely related with the tree topology, remaining consistent (Figure 2a–d). IKOV and LLEBV were located on a clade separate from MBLV and WCBV in all phylogenetic trees and may constitute a separate phylogroup (Figure 2a–d). Interestingly, the topology of the clade containing PBLV differed depending on the genomic region analyzed. Phylogenetic analysis based on the N gene suggests that PBLV was most closely related to TWBLV-1 but was also closely related to DUVV, EBLV-1, and TWBLV-2 (Figure 2a); however, a change in the topology occurred in the phylogenetic analysis based on the G gene, and PBLV grouped with DUVV, EBLV-1, and IRKV (Figure 2b). Phylogenetic analysis based on the concatenated N + P + M + G + L CDS and complete genome sequence suggests that PBLV was most closely related to TWBLV-1 and TWBLV-2 but was located on a separate branch (Figure 2c,d). Based on phylogenetic inference, PBLV is closely related, yet distinct from TWBLV-1 and -2. 

### 3.3. Pathogenic Determinants

Various changes that affect the coding regions of lyssaviruses have been described to alter their pathogenicity and/or play a role in immune evasion. A summary of these changes in MBLV and PBLV compared to RABV and WCBV is provided in Table 3. A summary of the pathogenic determinants for all representative lyssaviruses is provided in Table S3. It is important to note that the majority of these changes and their impact on pathogenesis were assessed for RABV and extrapolated to other lyssaviruses. F273, Y394, and F395 in the N CDS were important for viral pathogenesis and the evasion of the RIG-I-mediated immune response [48] and were conserved in PBLV. However, similar to WCBV, MBLV contained amino acid changes at positions 394 and 395 (Y394F and F395Y), which were associated with reduced pathogenicity for RABV [48]. D143 and Q147 in the P CDS allowed for the interaction of the viral P protein with LC8, which was important for retrograde intracellular virus transport [49] and was conserved in MBLV and PBLV. 

The PSAP motif in the M CDS of vesicular stomatitis virus, a virus that also belongs to the *Rhabdoviridae* family, was important for pathogenesis, and the disruption of the motif resulted in an attenuated phenotype [50]. While the PSAP motif was conserved in MBLV, PBLV contained a P22I mutation. The PPEY motif at residues 35–38 in the M CDS was important for virion release and pathogenesis [51] and was conserved in MBLV and PBLV. R77 and E81 in the M CDS enabled mitochondrial disruption and the induction of apoptosis [52]. In MBLV and PBLV, R77 was conserved and an E81G mutation was present. A V95A mutation in the M CDS increased cytopathic effect, which was partly due to an increase in apoptosis [53]. V95 was conserved in MBLV and PBLV. 

The majority of the pathogenic determinants have been identified in the G ectodomain. A K83R mutation in the G CDS resulted in decreased pathogenicity due to decreased G protein expression, and increased apoptosis and blood–brain barrier (BBB) permeability [54]; however, K83 was conserved in MBLV and PBLV. An N194K, not N194S, mutation was important for pathogenicity and resulted in increased virus spread, faster internalization, and a shift in the pH threshold for membrane fusion [55]. While N194 was conserved in PBLV, an N194T change was present in MBLV. R196 was conserved in MBLV and PBLV, which enabled viral activity with the nicotinic acetylcholine receptors [56]. A242, D255, and I268 enabled efficient cell-to-cell spread [57], and both MBLV and PBLV contained mutations at more than one of these amino acid positions (MBLV: A242S, D255S, and I268L; PBLV: A242S and I268L). F318S/V and H352Y/R mutations in the G CDS abolished the interaction of the viral G protein with the neurotrophin receptor (p75NTR) [58]. MBLV contained F318I and H352Y mutations, whereas PBLV contained F318M and H352Y mutations. K330 and R333, located at antigenic site III, played a role in pathogenesis, with double mutants, K330N and R333M, being avirulent [59]. Single mutants suggest that R333 was important for pathogenesis with R333M/Q/G mutations, resulting in an avirulent or attenuated phenotype [59,60], whereas a K330N mutant retained its virulence [59]. While both positions were conserved in PBLV, MBLV, similar to WCBV, contained K330I and R333E mutations. G349 in the G CDS was conserved in MBLV and PBLV, which was important for pathogenicity [61]. K1685 and K1829 in the L CDS played a role in pathogenicity and immune evasion [62] and were conserved in MBLV and PBLV.

### 3.4. Antigenic Determinants

**Figure 3:**
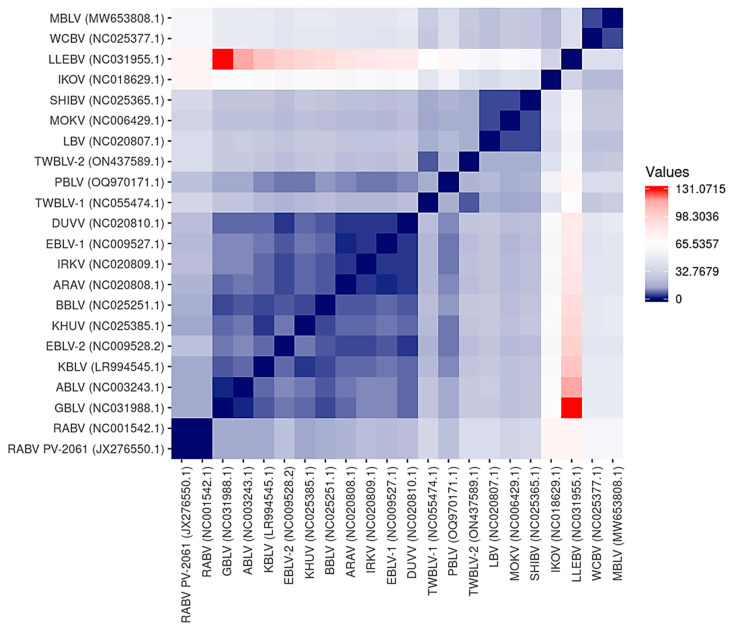
Heatmap illustrating the antigenic distance estimations between representative lyssaviruses. Antigenic distance estimations were calculated to demonstrate the similarity among the antigenic sites on the G protein among representative lyssaviruses. Lyssaviruses with identical antigenic sites are represented by a score of zero, which is indicated in dark blue. An increase in the score represents more dissimilar antigenic sites, as indicated by the scale. The most dissimilar antigenic distance estimates are indicated in red.

The antigenic sites identified on the lyssavirus G protein ectodomain are responsible for differential neutralization profiles among lyssaviruses [63]. A summary of the antigenic sites II-b (34–42), II-a (198–200), I (226–331), IV (251), G5 (261–264), III (330–338), and G1 (342–343) is provided for MBLV and PBLV, and representative lyssaviruses in Table S4 and a visual representation based on the overall antigenic distance estimates is provided in Figure 3. The antigenic sites of MBLV and WCBV were highly similar and identical for sites II-a, I, G5, and G1, and highly similar for sites II-b (Y34D, T37S) and III (E337D, V338I). Cross-neutralization between MBLV and WCBV is, therefore, highly likely; however, the MBLV antigenic sites were significantly different from the RABV PV-2061 strain. While PBLV contained antigenic sites that were identical to those of RABV PV-2061 (sites II-a, IV, and G1), some antigenic sites were not shared by any other lyssaviruses (sites II-b, G5, and III), and the degree of cross-neutralization will need to be experimentally determined. For PBLV, site I was identical to GBLV, ABLV, KBLV, EBLV-2, TWBLV-1, and TWBLV-2, site II-b was highly similar to EBLV-1 and DUVV (G34E), and ARAV (A40P), and site III was highly similar to EBLV-2 (T336K, V338I) and KHUV (S336K, E337D). The antigenic distance estimates demonstrate that MBLV and WCBV were antigenically highly similar but dissimilar to LLEBV and IKOV, and all other lyssaviruses, whereas PBLV was antigenically more similar to phylogroup I lyssaviruses than phylogroup II or the unassigned lyssaviruses. The antigenic distance estimates suggest that antigenically, PBLV was most similar to KBLV and that phylogroup I lyssaviruses were antigenically highly similar, but that RABV, TWBLV-1, and TWBLV-2 were antigenically more dissimilar to other phylogroup I lyssaviruses. The highest antigenic distance estimate was obtained between GBLV and LLEBV and suggests that these lyssaviruses were antigenically highly dissimilar.

### 3.5. Evaluation of Ecological Niche

**Figure 4:**
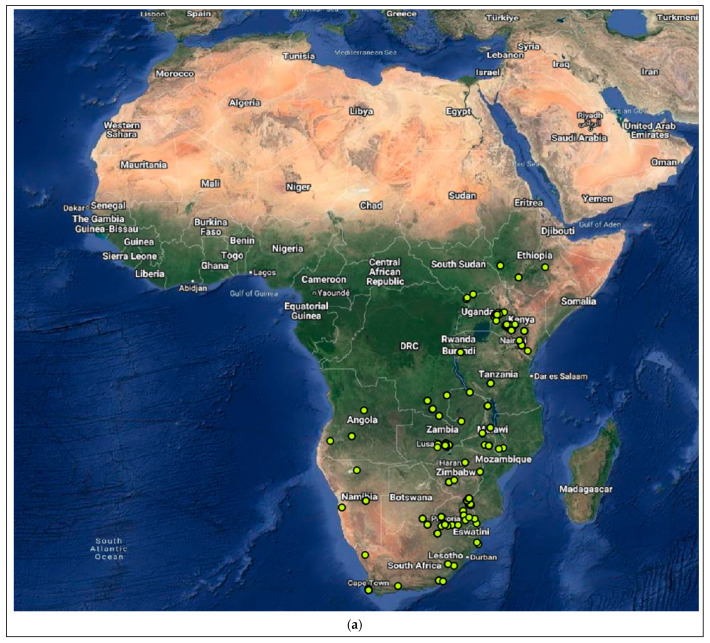

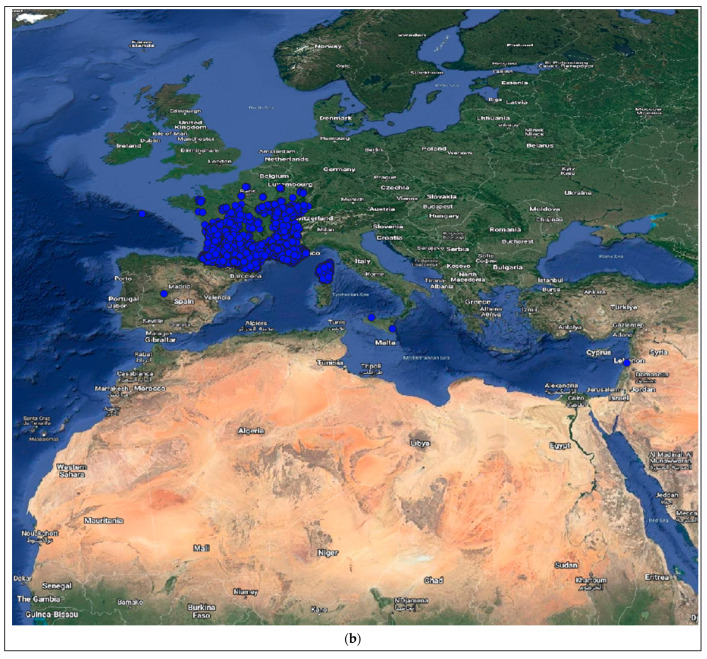

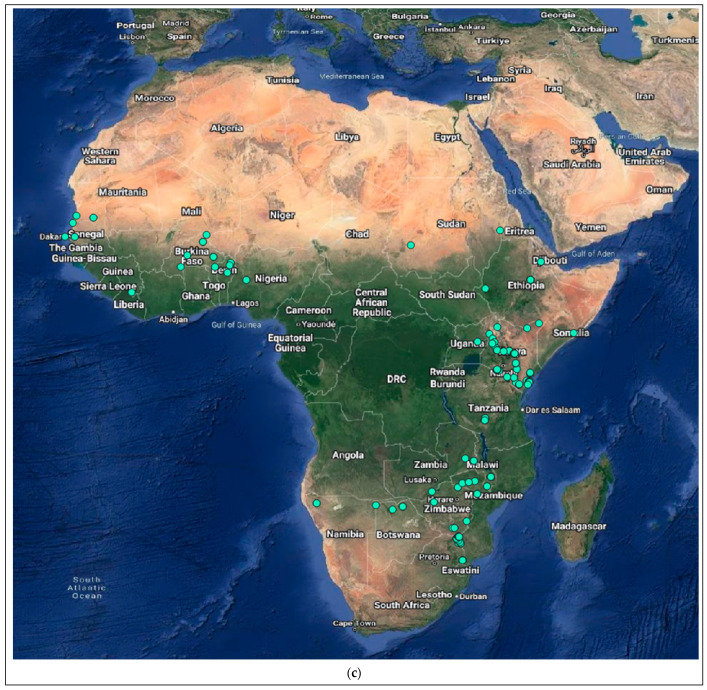

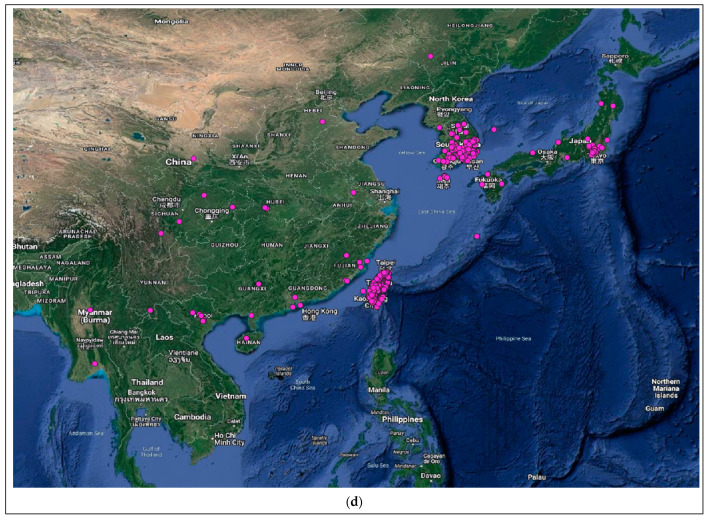

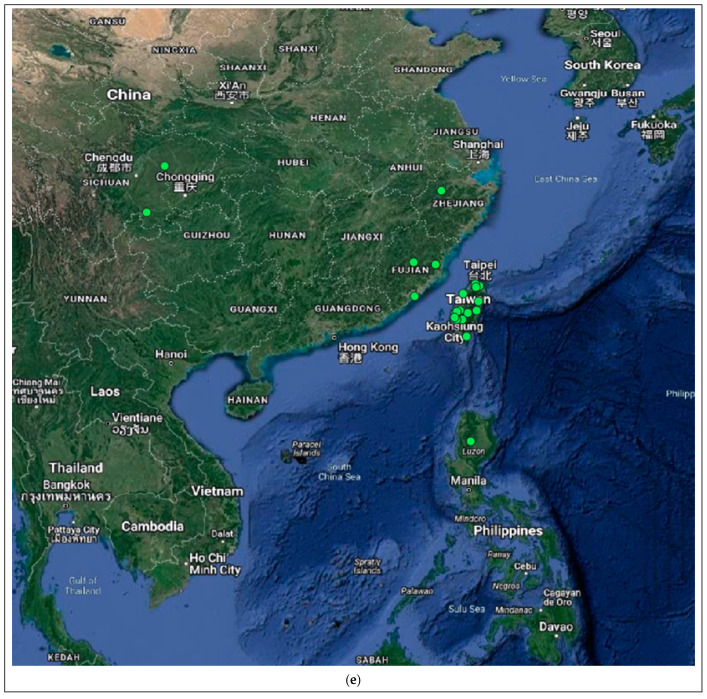
Distribution of the host species of MBLV and PBLV, and the most closely related lyssaviruses to MBLV and PBLV. Distribution maps for (**a**) *M. natalensis* (host of MBLV), (**b**) *M. schreibersii* (host of WCBV; most closely related virus to MBLV), (**c**) *N. schlieffeni* (host of PBLV), (**d**) *P. abramus* (host of TWBLV-1; most closely related to PBLV) and (**e**) *N. p. velutinus* (host of TBWLV-2; most closely related to PBLV) based on available georeferenced records.

The *Miniopterus* and *Nycticeinops* genera previously belonged to the family *Vespertilionidae* in the subfamilies *Miniopterinae* and *Vespertilioninae*, respectively; however, due to significant morphological, embryological, immunological, and genetic differences between miniopterine bats and other vespertilionids, the *Miniopterinae* subfamily was elevated to full family status and no longer form part of the family *Vespertilionidae* [64]. Significant differences were demonstrated between *M. natalensis*, previously a subspecies of *M. schreibersii*, and *M. schreibersii* [65,66,67], and subsequently, *M. natalensis* was elevated to full species rank after phylogenetic analysis confirmed that these two species are genetically distinct [67]. *M. natalensis* and *N. schlieffeni* have a primarily African distribution with limited records from the Arabian Peninsula, including Saudi Arabia and Yemen [68,69,70,71,72]. The mapping of georeferenced records for *M. natalensis* suggest a widespread distribution, particularly in southern and eastern Africa, and suggest its presence in at least Angola, Botswana, Burundi, the Democratic Republic of the Congo, Ethiopia, Kenya, Lesotho, Malawi, Mozambique, Namibia, South Africa, Tanzania, Uganda, Zambia, and Zimbabwe (Figure 4a), whereas the distribution of *M. schreibersii*, the host species of the most closely related virus to MBLV, is largely confined to Europe (Figure 4b). However, due to the frequent misidentification of *M. natalensis* and *M. schreibersii*, the revision of records may be required to confirm its distribution. In addition, *M. arenarius*, previously considered a subspecies of *M. natalensis* described in East Africa, is likely a distinct species [73]. *M. natalensis* was listed as a species of least concern by the International Union for Conservation of Nature (IUCN) in 2017 due to its wide distribution and presumed large population numbers and is considered to be unlikely to decline fast enough to justify listing it in a more threatened category [74]. The mapping of georeferenced records for *N. schlieffeni* suggests a widespread but disjunct distribution and suggests its presence in at least Benin, Botswana, Burkina Faso, Ethiopia, Guinea, Kenya, Malawi, Mauritania, Mozambique, Namibia, Nigeria, Senegal, Somalia, South Africa, Sudan, Tanzania, Uganda, Zambia, and Zimbabwe (Figure 4c). However, the presence of *N. schlieffeni* has also been reported in Angola, Cameroon, Central African Republic, Chad, Côte d’Ivoire, the Democratic Republic of the Congo, Djibouti, Egypt, Eritrea, Eswatini, Ghana, Mali, Niger, and Togo [41,75,76,77,78,79,80,81,82,83], whereas the distribution of *P. abramus* and *N. p. velitunus*, the host species of the most closely related viruses to PBLV, is largely confined to eastern Asia (Figure 4d,e). *N. schlieffeni* was listed as a species of least concern by the IUCN in 2017 due to its wide distribution and lack of major threats [84]. 

*M. natalensis* can thermoregulate between 5 and 40 °C [85] and roosts in caves in large numbers [86,87]. In Kruger National Park in South Africa, *M. natalensis* also roosts in rock crevices and the lofts of houses [88]; however, caves are crucial due to their use as night-roosting sites [86]. *M. natalensis* are insectivorous bats and clutter-edge foragers that feed on insects that belong to the orders Diptera, Hemiptera, Coleoptera, Lepidoptera, Hymenoptera, Isoptera, and Trichoptera [67,86,89,90,91,92]. 

*N. schlieffeni* are non-migratory and roost alone or in small groups in the crevices and hollows of trees during the day but can also utilize rock crevices, huts, houses, and cellars; however, they have been reported to roost in large numbers in rock crevices in Kruger National Park in South Africa [88,89,93,94,95]. *N. schlieffeni* are insectivorous, clutter-edge foragers that primarily feed on beetles (order Coleoptera) in excess of their proportional abundance, and to a lesser extent, insects that belong to the orders Diptera, Lepidoptera, Hemiptera, Hymenoptera, and Trichoptera [89,96,97]. 

## 4. Discussion

Biosurveillance for lyssaviruses, particularly in bat populations, is important to identify novel and/or possibly zoonotic lyssaviruses to improve our understanding of lyssavirus diversity, ecology, epidemiology, and host-species associations, which, in turn, will inform risk assessments and mitigation strategies for the prevention of rabies. However, biosurveillance is inadequate in large parts of the world, including Africa, and is required to explore the diversity, distribution, and genetic diversity of lyssaviruses. In this paper, we describe the taxonomic features of MBLV and PBLV and evaluate various aspects of the ICTV criteria for lyssavirus species demarcation. 

The analysis of the nucleotide and amino acid sequence identities and the phylogenetic analysis are in agreement that MBLV and PBLV group with the unassigned and phylogroup I lyssaviruses, respectively. While the nucleotide and amino acid identities suggest that PBLV is most closely related to EBLV-1, the phylogenetic analysis confirms that PBLV is closely related to DUVV, EBLV-1, TWBLV-1, and TWBLV-2 but is most closely related to, yet distinct from, TWBLV-1 and TWBLV-2. The phylogenetic analysis suggests that MBLV is most closely related to WCBV; however, LLEBV and IKOV were represented in a separate clade, which suggests that the unassigned lyssaviruses may constitute two separate phylogroups. In addition, the antigenic distance estimates were high between LLEBV and IKOV, and MBLV and WCBV, suggesting that these viruses are antigenically dissimilar.

Current rabies biologicals are based on the Pasteur vaccine (PV) and PV-derivates and provide protection against phylogroup I but not phylogroup II lyssaviruses or the unassigned lyssaviruses [5,98,99,100]. The degree of cross-neutralization is thought to be a predictor for the level of protection that is offered by rabies prophylaxis, and the exchange of the antigenic sites among lyssaviruses from different phylogroups confirmed the importance of the antigenic sites in cross-neutralization [63]. However, it is important to note that this study suggested that some antigenic sites may be more important for cross-neutralization than others and that it may differ for lyssaviruses from different phylogroups [63]. The analysis of the antigenic sites suggests that the antigenic sites for MBLV are highly similar to WCBV, but not RABV PV-2061, LLEBV, or IKOV, and cross-neutralization with WCBV is, therefore, likely. While PBLV contained antigenic sites that were identical or highly similar to RABV PV-2061, some antigenic sites that have previously been demonstrated to be important for cross-neutralization (sites II-b, G5, and III) were significantly different. Antigenic relationships cannot be based solely on genetic analysis since a substantial difference exists for some lyssaviruses between the genetic and antigenic distances [101]. In vitro and in vivo studies are required to determine the degree of cross-neutralization and cross-protection conferred by rabies biologicals against novel lyssaviruses [5,98,99,100].

Pathogenic determinants that impact pathogenesis and the evasion of the immune response have been identified and described; however, these determinants were analyzed independently, and the cumulative effect on the phenotype can only be determined experimentally. While the majority of the pathogenic determinants were conserved in MBLV and PBLV, some amino acid changes previously shown to reduce pathogenicity were identified for MBLV. In addition, some uncharacterized changes were identified, for which the likely impact on the phenotype is unknown but may affect mitochondrial disruption, the induction of apoptosis, cell-to-cell spread, and interaction with the neurotrophin receptor [52,55,57,58]. For MBLV, two sites that are important for pathogenesis and cross-neutralization by neutralizing antibodies that target antigenic site III [59], were mutated and may result in reduced pathogenicity and a lack of cross-neutralization with RABV. However, further investigation is required to determine the impact of these changes on pathogenesis and the evasion of the immune response. At the time of writing this article, attempts to isolate PBLV were unsuccessful, which may, in part, be due to a discontinuous supply of electricity at the rehabilitation center, which resulted in several freeze–thaw cycles before receipt of the bat for investigation or attempts to isolate PBLV in cell culture rather than mice. However, due to the availability of the complete genome sequence for PBLV, the impact of changes in the antigenic and pathogenic determinants can be assessed in the future using reverse genetics systems developed for RABV.

The majority of lyssaviruses appear to have co-evolved with a limited number of reservoir species and are geographically restricted, with their distribution reflecting that of their reservoir species [15]. MBLV was identified in two apparently healthy *M. natalensis* bats, whereas PBLV was identified in an *N. schlieffeni* bat with neurological signs. Based on an assessment of the host species for MBLV and PBLV, and their known geographical distribution, MBLV and PBLV possibly occupy distinct ecological niches. *M. natalensis* and *N. schlieffeni* are widely distributed across Africa, with limited records from the Arabian Peninsula [68,69,70,71,72], and there is no overlap in the distribution of the host species of WCBV (*M. schreibersii*) and, TWBLV-1 (*P. abramus*) and TWBLV-2 (*N. p. velutinus*), the most closely related lyssaviruses to MBLV and PBLV, respectively (Figure 4a–e). *M. natalensis* are migratory bats that frequently roost in large numbers in caves, require an open water source near the roost, and forage in the open spaces above the water [86,87], whereas *N. schlieffeni* are non-migratory bats that frequently roost in the crevices of trees in smaller numbers, are considered opportunistic feeders due to their maneuverability, and restrict their activities near human settlements to gain access to water, roosts, and/or prey [89,97,102]. While significant overlap in the diet of sympatric insectivorous bats has been demonstrated, evidence of spatial resource partitioning by the use of different foraging strategies, habitat use, and the consumption of different proportions of prey with little to no temporal resource partitioning allow various insectivorous bat species to co-exist in a shared habitat with shared food sources [89,97]. 

The ICTV criteria for the demarcation of new lyssavirus species include the following: (a) has a nucleotide identity of 78–80% or 80% for the N gene or concatenated N + P + M + G + L CDS, respectively; (b) can be phylogenetically differentiated from other established lyssavirus species; (c) can be serologically distinguished by virus-neutralization tests; (d) occupies a distinct ecological niche, as evidenced by the host or vector species, geographical range, or pathobiological properties. PBLV had a nucleotide identity of 67.70–74.56% for the N gene and 63.14–75.55% for the concatenated N + P + M + G + L CDS, was phylogenetically distinguishable from other lyssaviruses, and occupied a distinct ecological niche, as evidenced by the host species. In addition, the antigenic similarity scores suggest that PBLV will likely be serologically distinguishable from other lyssaviruses and may, therefore, represent a new lyssavirus species. MBLV had a nucleotide identity of 67.18–77.52% for the N gene and 64.21–78.94% for the concatenated N + P + M + G + L CDS, could not be phylogenetically distinguished from WCBV and occupied a distinct ecological niche, as evidenced by the host species. The antigenic similarity scores for antigenic regions suggest that WCBV and MBLV are highly similar and may not be serologically distinguishable. MBLV is, therefore, related to WCBV but may not represent a new lyssavirus species; however, cross-neutralization tests will be required to confirm whether MBLV and WCBV are serologically distinguishable. We will continue to attempt virus isolation for PBLV and will perform experimental verification of cross-neutralization between MBLV and PBLV and selected lyssavirus isolates to address the ICTV criteria for lyssavirus species demarcation.

## Figures and Tables

**Table 1:** Genome characteristics of MBLV and PBLV.

	3′ UTR	N Gene (CDS)	N-P IGS	P Gene (CDS)	P-M IGS	M Gene (CDS)	M-G IGS	G Gene (CDS)	G-L IGS	L Gene (CDS)	5′ UTR
Lyssaviruses	58 nt	1395–1443 nt (1353–1356 nt, 450–451 aa)	2–4 nt	957–1042 nt (894–918 nt, 297–305 aa)	2–5 nt	768–806 nt (609 nt, 202 aa)	5–39 nt	2047–2304 nt (1569–1629 nt, 522–542 aa)	19–101 nt	6454–6510 nt (6381–6429 nt, 2126–2142 aa)	42–71 nt
MBLV (MW653808.1)	58 nt	1395 nt (1353 nt, 450 aa)	4 nt	1041 nt (894 nt, 297 aa)	2 nt	768 nt (609 nt, 202 aa)	39 nt	2304 nt (1578 nt, 525 aa)	101 nt	6509 nt (6384 nt, 2127 aa)	57 nt
PBLV (OQ970171.1)	58 nt	1432 nt (1356 nt, 451 aa)	2 nt	989 nt (897 nt, 298 aa)	5 nt	807 nt (609 nt, 202 aa)	5 nt	2115 nt (1596 nt, 531 aa)	20 nt	6477 nt (6384 nt, 2127 aa)	68 nt

The length of each genome element is provided, including the range for all known lyssaviruses. For coding sequences (CDS), the length of the gene is provided, followed by the length of the coding sequence and amino acid sequence in brackets.

**Table 2:** Transcription initiation and termination signals of MBLV and PBLV.

	N Gene TIS/TTS	P Gene TIS/TTS	M Gene TIS/TTS	G Gene TIS/TTS	L Gene TIS/TTS
MBLV (MW653808.1)	AACACCCCT	AACACCCCT	AACACCCCT	AACATCCCT	AACACCTCT
	TGAAAAAAA	TGAAAAAAA	TGAAAAAAA	TGAAAAAAA	TGAAAAAAA
PBLV (OQ970171.1)	AACACCCCT	AACACCACT	AACACCACT	AACAGCCCT	AACACCCCT
	TGAAAAAAA	TGAAAAAAA	TGAAAAAAA	TGAAAAAAA	TGAAAAAAA

The transcription initiation signal (TIS) and transcription termination signal (TTS) of each gene are provided.

**Table 3:** Summary of pathogenic determinants in RABV, PBLV, MBLV, and WCBV.

Amino Acid Position	N			P	M					G											L	
	F273	Y394	F395	DKSTQ 143–147	PSAP 22–25	PPEY 35–38	R77	E81	V95	K83	N194	R/K196	A242	D255	I268	F318	K330	R333	G349	H352	K1685	K1829
RABV (NC001542.1)					**V**SAP									**G**								
PBLV (OQ970171.1)				DKS**V**Q	ISAP			**G**					**S**		L	M				**Y**		
WCBV (NC025377.1)		**F**	**Y**	D**IAV**Q				**N**			T		**S**	**S**	L	I	**I**	**E**		**Y**		
MBLV (MW653808.1)		**F**	**Y**	D**IAI**Q				**G**			T		**S**	**S**	L	I	**I**	**E**		**Y**		

Amino acid changes are underlined and in bold if the change affects the characteristics of the amino acid.

## Data Availability

All relevant data are included within the manuscript and its Supplementary Materials.

## Supplementary Materials

The following supporting information can be downloaded at: https://www.mdpi.com/article/10.3390/v15102047/s1, Table S1. Percentage nucleotide and amino acid identity with MBLV; Table S2. Percentage nucleotide and amino acid identity with PBLV; Table S3. Summary of pathogenic determinants in lyssaviruses; Table S4. Summary of amino acid changes in antigenic regions on the G protein for lyssaviruses.
