# Supplementary material for: Evaluation of Taxonomic Characteristics of Matlo and Phala Bat Rabies-Related Lyssaviruses Identified in South Africa
Source: Viruses. 2023 Oct 4;15(10):2047. doi: 10.3390/v15102047 (PMC10611238; doi:10.3390/v15102047)
Supplement: Supplementary file 1 [file viruses-15-02047-s001.zip › viruses-2613254-supplementary.pdf]

Supplementary information

Table S1. Percentage nucleotide and amino acid identity with MBLV.

	N gene	P gene	M gene	G gene	L gene	Concatenated N+P+M+G+L CDS
**Phylogroup I**						
**RABV PV-2061 (JX276550.1)**	NT: 71.33%<br>AA: 81.78%	NT: 49.44%<br>AA: 43.25%	NT: 61.29%<br>AA: 76.24%	NT: 49.51%<br>AA: 50.96%	NT: 66.67%<br>AA: 74.57%	NT: 64.80%<br>AA: 69.61%
**RABV (NC001542.1)**	NT: 68.85%<br>AA: 81.78%	NT: 49.44%<br>AA: 43.25%	NT: 60.70%<br>AA: 75.25%	NT: 50.37%<br>AA: 50.77%	NT: 66.84%<br>AA: 74.57%	NT: 64.81%<br>AA: 69.53%
**GBLV (NC031988.1)**	NT: 67.87%<br>AA: 83.11%	NT: 50.05%<br>AA: 47.40%	NT: 63.89%<br>AA: 78.22%	NT: 48.87%<br>AA: 52.11%	NT: 67.94%<br>AA: 75.32%	NT: 65.54%<br>AA: 70.70%
**ABLV (NC003243.1)**	NT: 69.31%<br>AA: 82.00%	NT: 50.20%<br>AA: 45.67%	NT: 62.99%<br>AA: 71.78%	NT: 50.00%<br>AA: 52.11%	NT: 67.20%<br>AA: 74.71%	NT: 65.30%<br>AA: 69.75%
**KBLV (LR994545.1)**	NT: 68.36%<br>AA: 82.00%	NT: 50.94%<br>AA: 44.98%	NT: 62.24%<br>AA: 76.73%	NT: 49.78%<br>AA: 50.96%	NT: 68.12%<br>AA: 76.59%	NT: 65.65%<br>AA: 70.90%
**EBLV-2 (NC009528.2)**	NT: 67.18%<br>AA: 80.89%	NT: 51.58%<br>AA: 46.02%	NT: 62.89%<br>AA: 77.23%	NT: 49.93%<br>AA: 50.38%	NT: 68.06%<br>AA: 76.07%	NT: 65.89%<br>AA: 70.50%
**KHUV (NC025385.1)**	NT: 69.11%<br>AA: 81.56%	NT: 49.53%<br>AA: 45.33%	NT: 62.58%<br>AA: 78.71%	NT: 49.75%<br>AA: 51.15%	NT: 67.94%<br>AA: 76.26%	NT: 65.61%<br>AA: 70.84%
**BBLV (NC025251.1)**	NT: 69.96%<br>AA: 82.00%	NT: 49.85%<br>AA: 46.37%	NT: 62.24%<br>AA: 77.72%	NT: 48.79%<br>AA: 51.34%	NT: 68.10%<br>AA: 75.93%	NT: 65.81%<br>AA: 70.81%
**ARAV (NC020808.1)**	NT: 69.50%<br>AA: 83.56%	NT: 50.51%<br>AA: 47.75%	NT: 63.02%<br>AA: 77.23%	NT: 48.61%<br>AA: 50.77%	NT: 68.26%<br>AA: 76.12%	NT: 65.77%<br>AA: 71.04%
**IRKV (NC020809.1)**	NT: 68.88%<br>AA: 83.56%	NT: 52.05%<br>AA: 44.83%	NT: 65.49%<br>AA: 77.72%	NT: 49.13%<br>AA: 51.62%	NT: 67.58%<br>AA: 75.79%	NT: 65.79%<br>AA: 71.03%
**EBLV-1 (NC009527.1)**	NT: 68.47%<br>AA: 82.44%	NT: 50.82%<br>AA: 45.21%	NT: 64.10%<br>AA: 77.23%	NT: 48.48%<br>AA: 50.00%	NT: 68.22%<br>AA: 75.88%	NT: 65.81%<br>AA: 70.58%
**DUVV (NC020810.1)**	NT: 69.92%<br>AA: 84.22%	NT: 50.51%<br>AA: 44.14%	NT: 64.03%<br>AA: 76.24%	NT: 48.13%<br>AA: 49.43%	NT: 68.29%<br>AA: 75.41%	NT: 66.03%<br>AA: 70.29%
**TWBLV-1 (NC055474.1)**	NT: 70.17%<br>AA: 83.56%	NT: 49.33%<br>AA: 42.07%	NT: 62.92%<br>AA: 78.71%	NT: 47.16%<br>AA: 48.28%	NT: 67.20%<br>AA: 75.98%	NT: 65.22%<br>AA: 70.37%
**TWBLV-2 (ON437589.1)**	NT: 68.47%<br>AA: 84.00%	NT: 51.18%<br>AA: 45.67%	NT: 62.66%<br>AA: 77.23%	NT: 47.67%<br>AA: 47.89%	NT: 67.74%<br>AA: 75.83%	NT: 65.68%<br>AA: 70.53%
**PBLV (OQ970171.1)**	NT: 67.79%	NT: 51.33%	NT: 62.37%	NT: 48.75%	NT: 67.85%	NT: 65.79%

	AA: 82.89%	AA: 48.28%	AA: 79.21%	AA: 50.77%	AA: 75.98%	AA: 71.06%
**Phylogroup II**						
**LBV (NC020807.1)**	NT: 70.10%<br>AA: 83.33%	NT: 49.80%<br>AA: 44.37%	NT: 64.91%<br>AA: 75.25%	NT: 50.65%<br>AA: 54.60%	NT: 67.57%<br>AA: 75.65%	NT: 66.03%<br>AA: 70.92%
**MOKV (NC006429.1)**	NT: 67.93%<br>AA: 83.56%	NT: 50.97%<br>AA: 46.42%	NT: 63.25%<br>AA: 76.24%	NT: 52.22%<br>AA: 53.07%	NT: 67.93%<br>AA: 75.04%	NT: 65.96%<br>AA: 70.56%
**SHIBV (NC025365.1)**	NT: 69.88%<br>AA: 83.56%	NT: 51.06%<br>AA: 46.08%	NT: 64.44%<br>AA: 78.71%	NT: 50.55%<br>AA: 53.45%	NT: 68.70%<br>AA: 76.59%	NT: 66.69%<br>AA: 71.71%
**Unassigned lyssaviruses**						
**IKOV (NC018629.1)**	NT: 68.07%<br>AA: 79.56%	NT: 55.26%<br>AA: 48.08%	NT: 61.14%<br>AA: 73.27%	NT: 49.59%<br>AA: 48.85%	NT: 65.62%<br>AA: 71.97%	NT: 64.21%<br>AA: 67.71%
**LLEBV (NC031955.1)**	NT: 69.00%<br>AA: 81.56%	NT: 51.95%<br>AA: 46.69%	NT: 64.16%<br>AA: 75.25%	NT: 48.96%<br>AA: 47.79%	NT: 65.73%<br>AA: 72.81%	NT: 64.21%<br>AA: 68.30%
**WCBV (NC025377.1)**	NT: 77.52%<br>AA: 95.56%	NT: 71.28%<br>AA: 83.16%	NT: 84.90%<br>AA: 96.53%	NT: 70.11%<br>AA: 86.67%	NT: 78.39%<br>AA: 92.76%	NT: 78.94%<br>AA: 91.64%

Table S2. Percentage nucleotide and amino acid identity with PBLV.

	N gene	P gene	M gene	G gene	L gene	Concatenated N+P+M+G+L CDS
**Phylogroup I**						
**RABV PV-2061 (JX276550.1)**	NT: 73.25%<br>AA: 88.00%	NT: 60.75%<br>AA: 58.92%	NT: 67.46%<br>AA: 82.18%	NT: 56.81%<br>AA: 68.13%	NT: 71.81%<br>AA: 83.78%	NT: 70.98%<br>AA: 79.92%
**RABV (NC001542.1)**	NT: 70.98%<br>AA: 87.78%	NT: 60.65%<br>AA: 58.59%	NT: 65.84%<br>AA: 81.19%	NT: 56.63%<br>AA: 68.32%	NT: 71.76%<br>AA: 83.78%	NT: 70.96%<br>AA: 79.83%
**GBLV (NC031988.1)**	NT: 72.79%<br>AA: 89.33%	NT: 61.16%<br>AA: 61.82%	NT: 68.07%<br>AA: 84.16%	NT: 59.54%<br>AA: 69.01%	NT: 73.44%<br>AA: 85.94%	NT: 72.31%<br>AA: 81.76%
**ABLV (NC003243.1)**	NT: 71.64%<br>AA: 88.44%	NT: 58.98%<br>AA: 56.57%	NT: 66.67%<br>AA: 80.20%	NT: 57.14%<br>AA: 67.62%	NT: 72.31%<br>AA: 84.91%	NT: 70.96%<br>AA: 80.23%
**KBLV (LR994545.1)**	NT: 72.64%<br>AA: 87.36%	NT: 61.97%<br>AA: 61.28%	NT: 71.48%<br>AA: 85.15%	NT: 62.80%<br>AA: 72.81%	NT: 73.77%<br>AA: 88.15%	NT: 73.02%<br>AA: 83.46%
**EBLV-2 (NC009528.2)**	NT: 72.05%<br>AA: 86.92%	NT: 60.37%<br>AA: 59.26%	NT: 69.49%<br>AA: 82.67%	NT: 61.38%<br>AA: 73.09%	NT: 73.45%<br>AA: 86.98%	NT: 72.49%<br>AA: 82.42%
**KHUV (NC025385.1)**	NT: 72.08%<br>AA: 88.47%	NT: 62.55%<br>AA: 61.28%	NT: 72.53%<br>AA: 86.63%	NT: 60.98%<br>AA: 70.72%	NT: 73.85%<br>AA: 88.06%	NT: 72.76%<br>AA: 83.29%
**BBLV (NC025251.1)**	NT: 72.86%<br>AA: 89.36%	NT: 62.17%<br>AA: 61.95%	NT: 70.98%<br>AA: 87.13%	NT: 59.98%<br>AA: 71.18%	NT: 74.31%<br>AA: 87.45%	NT: 73.15%<br>AA: 83.23%
**ARAV (NC020808.1)**	NT: 73.94%<br>AA: 90.91%	NT: 62.67%<br>AA: 64.31%	NT: 74.84%<br>AA: 87.62%	NT: 61.74%<br>AA: 71.86%	NT: 74.27%<br>AA: 87.73%	NT: 73.40%<br>AA: 83.85%
**IRKV (NC020809.1)**	NT: 71.72%<br>AA: 90.24%	NT: 65.12%<br>AA: 61.41%	NT: 73.33%<br>AA: 90.59%	NT: 60.99%<br>AA: 72.90%	NT: 75.06%<br>AA: 89.94%	NT: 73.82%<br>AA: 85.42%
**EBLV-1 (NC009527.1)**	NT: 74.56%<br>AA: 92.46%	NT: 65.93%<br>AA: 67.79%	NT: 74.75%<br>AA: 89.60%	NT: 65.49%<br>AA: 74.62%	NT: 76.16%<br>AA: 91.26%	NT: 75.55%<br>AA: 86.92%
**DUVV (NC020810.1)**	NT: 72.70%<br>AA: 90.91%	NT: 62.99%<br>AA: 63.09%	NT: 73.09%<br>AA: 89.60%	NT: 60.68%<br>AA: 71.56%	NT: 74.81%<br>AA: 88.67%	NT: 73.70%<br>AA: 84.45%
**TWBLV-1 (NC055474.1)**	NT: 73.46%<br>AA: 91.80%	NT: 63.87%<br>AA: 63.76%	NT: 71.93%<br>AA: 90.59%	NT: 59.23%<br>AA: 68.93%	NT: 75.14%<br>AA: 89.19%	NT: 73.69%<br>AA: 84.51%
**TWBLV-2 (ON437589.1)**	NT: 72.93%<br>AA: 90.91%	NT: 64.87%<br>AA: 64.98%	NT: 74.53%<br>AA: 90.59%	NT: 62.14%<br>AA: 69.98%	NT: 75.35%<br>AA: 90.50%	NT: 74.18%<br>AA: 85.44%
**Phylogroup II**						
**LBV (NC020807.1)**	NT: 70.84%<br>AA: 86.44%	NT: 51.79%<br>AA: 44.11%	NT: 64.42%<br>AA: 77.72%	NT: 52.38%<br>AA: 56.70%	NT: 71.17%<br>AA: 81.01%	NT: 68.57%<br>AA: 74.82%
**MOKV (NC006429.1)**	NT: 68.34%<br>AA: 84.00%	NT: 51.44%<br>AA: 42.91%	NT: 64.97%<br>AA: 76.73%	NT: 53.07%<br>AA: 57.09%	NT: 70.42%<br>AA: 79.78%	NT: 68.03%<br>AA: 73.73%

**SHIBV (NC025365.1)**	NT: 71.39%<br>AA: 86.67%	NT: 53.51%<br>AA: 46.28%	NT: 66.88%<br>AA: 80.20%	NT: 52.30%<br>AA: 57.09%	NT: 70.81%<br>AA: 81.48%	NT: 68.57%<br>AA: 75.52%
**Unassigned lyssaviruses**						
**IKOV (NC018629.1)**	NT: 67.70%<br>AA: 76.67%	NT: 49.89%<br>AA: 40.49%	NT: 59.80%<br>AA: 73.27%	NT: 46.83%<br>AA: 45.96%	NT: 65.36%<br>AA: 70.84%	NT: 63.14%<br>AA: 65.69%
**LLEBV (NC031955.1)**	NT: 67.76%<br>AA: 78.00%	NT: 51.05%<br>AA: 42.05%	NT: 63.73%<br>AA: 74.75%	NT: 47.56%<br>AA: 45.30%	NT: 65.82%<br>AA: 72.91%	NT: 63.87%<br>AA: 67.01%
**WCBV (NC025377.1)**	NT: 68.62%<br>AA: 83.33%	NT: 49.28%<br>AA: 47.24%	NT: 63.28%<br>AA: 78.71%	NT: 50.70%<br>AA: 49.43%	NT: 67.53%<br>AA: 76.45%	NT: 65.73%<br>AA: 71.04%
**MBLV (MW653808.1)**	NT: 67.79%<br>AA: 82.89%	NT: 51.33%<br>AA: 48.28%	NT: 62.37%<br>AA: 79.21%	NT: 48.75%<br>AA: 50.77%	NT: 67.85%<br>AA: 75.98%	NT: 65.79%<br>AA: 71.06%

Table S3. Summary of pathogenic determinants in lyssaviruses.

Amino acid position	N			P	M					G										L	
	F273	Y394	F395	DKSTQ 143-147	PSAP 22-25	PPEY 35-38	R77	E81	V95	K83	N194	R/K196	A242	D255	I268	F318	K330	R333	G349	H352	K1685	K1829
**Phylogroup I**																						
RABV PV-2061 (JX276550.1)					**V**SAP									**G**								
RABV (NC001542.1)					**V**SAP									**G**								
GBLV (NC031988.1)		**F**			**V**SAP						T		**S**			V			**E**	**Y**		
ABLV (NC003243.1)			L	**S**KSTQ	AS**TL**		**E**				**M**		**S**	**N**		I			**E**			
KBLV (LR994545.1)		**F**			ASAP						T		**S**	**N**		I				**Y**		
EBLV-2 (NC009528.2)		**L**	**Y**		**V**SAP			**G**			T		**S**		V	I				**Y**		
KHUV (NC025385.1)		**F**			**V**SAP	PPE**S**	K	**G**			T		**S**			I				**Y**		
BBLV (NC025251.1)		**F**			MSAP			**G**			T		**S**		V	I				**Y**		
ARAV (NC020808.1)		**F**			**V**SAP		K	**G**		R	T		**S**	**N**		I				**Y**		
IRKV (NC020809.1)		**F**		EKSTQ	**V**SAP		K	**G**			T		**S**	**G**		I						
EBLV-1 (NC009527.1)		**F**		DKST**R**	**V**SAP		**S**	**G**			T		**S**			I						
DUVV (NC020810.1)		**F**			**V**SAP			**G**		R	**R**	**M**	**S**			I				**Y**		
TWBLV-1 (NC055474.1)		**F**		DK**Y**TQ	**V**SAP		K	**G**					**S**	**G**		I	R					
TWBLV-2 (ON437589.1)		**F**			**V**SAP		K	**G**			T		**S**			I						
PBLV (OQ970171.1)				DKS**V**Q	ISAP			**G**					**S**		L	M				**Y**		
**Phylogroup II**																						
LBV (NC020807.1)		**F**		**N**RQTQ	ASAP		K	**N**	I		T	**T**	**S**			L		**D**	**N**	**M**		

MOKV (NC006429.1)	**F**		**SI**Q**I**Q	ASAP	K	**N**			S	**N**	**S**	**N**		L		**D**	**Q**	**M**
SHIBV (NC025365.1)	**F**		**N**KS**V**Q		K	**N**			T	**T**	**S**	**N**		A		**D**	**D**	**L**
**Unassigned lyssaviruses**																		
IKOV (NC018629.1)	**D**	**W**	DK**F**SQ	A**P**V**L**		**G**	I	**Q**	**A**	**T**	**S**	**S**		I		**D**		
LLEBV (NC031955.1)	**N**	**W**	DK**A**TQ	ASAP	K	**S**		**Q**	S	**T**	**S**	**S**	L	I		**S**		**Y**
WCBV (NC025377.1)	**F**	**Y**	D**IAV**Q			**N**			T		**S**	**S**	L	I	**I**	**E**		**Y**
MBLV (MW653808.1)	**F**	**Y**	D**IAI**Q			**G**			T		**S**	**S**	L	I	**I**	**E**		**Y**

Amino acid changes are underlined and in bold if the change affects the characteristics of the amino acid.

Table S4. Summary of amino acid changes in antigenic regions on the G protein for lyssaviruses.

Amino acid position	Site II-b	Site II-a	Site I	Site IV	Site G5	Site III	Site G1
	34-42	198-200	226-231	251	261-264	330-338	342-343
				**Phylogroup I**			
**RABV PV-2061 (JX276550.1)**	GCTNLSGFS[A]	KRA[D]	KLCGVL[G]	W[L]	HDFR[N]	KSVRTWNEI[R]	KG[T]
**RABV (NC001542.1)**	GCTNLSGFS[A]	KRA[D]	KLCGVL[G]	W[L]	HDFR[N]	KSVRTWNEI[R]	KG[T]
**GBLV (NC031988.1)**	GCTSLSGFS[B]	KKA[E]	KLCGI**S**[H]	W[L]	HDF**H**[O]	KSVR**A**WNEI	KG[T]
**ABLV (NC003243.1)**	GCTSLSGFS[B]	KKA[E]	KLCGI**S**[H]	W[L]	HDFN	KSVRTW**D**EI	KG[T]
**KBLV (LR994545.1)**	GCTTLS**A**FS	KRA[D]	KLCGI**S**[H]	W[L]	HDF**H**[O]	KSIR**D**WTEI	KG[T]
**EBLV-2 (NC009528.2)**	GCTTL**TV**FS	KKA[E]	KLCGI**S**[H]	W[L]	HDF**H**[O]	KSIR**E**WTDV	KG[T]
**KHUV (NC025385.1)**	GCTTLSGFT	KRA[D]	KLCGV**S**[I]	W[L]	HDF**H**[O]	KSIR**E**WSEI	KG[T]
**BBLV (NC025251.1)**	GCTNLSGFT	KKA[E]	KLCGV**S**[I]	W[L]	HDF**H**[O]	KSIRQWTEI	KG[T]
**ARAV (NC020808.1)**	GCTTLT**A**FS	KKA[E]	KLCGVM	W[L]	HDF**H**[O]	KSVR**E**WTEV	KG[T]
**IRKV (NC020809.1)**	GCTTLT**A**FN	KKA[E]	KLCGMA	W[L]	HDF**H**[O]	KSIR**E**W**K**EI[S]	KG[T]
**EBLV-1 (NC009527.1)**	GCTTLT**P**FS[C]	KKA[E]	RLCGV**P**	W[L]	HDF**H**[O]	KSVR**E**W**K**EV	KG[T]
**DUVV (NC020810.1)**	GCTTLT**P**FS[C]	KKA[E]	RLCGI**S**	W[L]	HDF**H**[O]	KSVR**E**W**K**EI[S]	KG[T]
**TWBLV-1 (NC055474.1)**	GCNTLS**S**FS	K**M**A	KLCGI**S**[H]	**P**	HDFR[N]	RSIRNWTEV	KG[T]
**TWBLV-2 (ON437589.1)**	GCNTLT**P**FS	**SM**A	KLCGI**S**[H]	Q	HDFR[N]	KSVRNWTEV	KG[T]
**PBLV (OQ970171.1)**	**E**CTTLT**P**FS	KRA[D]	KLCGI**S**[H]	W[L]	HSF**Q**	KSIR**E**W**K**DI	KG[T]
				**Phylogroup II**			
**LBV (NC020807.1)**	GCS**ET**S**S**FT	RKA	**T**LCG**KP**[J]	W[L]	H**NN**R[P]	K**R**V**D**NW**V**DI	KG[T]
**MOKV (NC006429.1)**	GCN**AE**S**S**FT	KKA[E]	**T**LCG**RP**	W[L]	H**ND**R	K**R**V**DK**W**A**DI	KG[T]
**SHIBV (NC025365.1)**	GCSS**S**S**T**FS	KKS	**T**LCG**KP**[J]	W[L]	H**NN**R[P]	K**R**V**DR**W**E**EI	KG[T]

**Unassigned lyssaviruses**							
**IKOV (NC018629.1)**	GCNEGSKVS	ILL	IICGKS	M	HTVK	KSVDNWTDI	PI[U]
**LLEBV (NC031955.1)**	NCTDHGEIN	RLF	TICGKS	V[M]	HTTK	KSVSNWSEI	PI[U]
**WCBV (NC025377.1)**	YCTTEQSIT	KLV[F]	SICGRQ[K]	V[M]	HDIK[Q]	IKVENWSEV	KG[T]
**MBLV (MW653808.1)**	DCTSEQSIT	KLV[F]	SICGRQ[K]	A	HDIK[Q]	IKVENWSDI	KG[T]

Amino acid changes from the amino acid sequence for RABV PV-2061 (JX276550.1) are underlined and highlighted if the change affects the characteristics of the amino acid.

Identical antigenic sites are indicated from A-U.
